# Recurrent angioedema manifestation and treatment response in two patients from different families caring the myoferlin gene mutation: case series

**DOI:** 10.1186/s13023-025-03932-9

**Published:** 2025-08-12

**Authors:** Daria S. Fomina, Marina S. Lebedkina, Elena N. Bobrikova, Yulia D. Yukhnovskaya, Anna A. Roppelt, Olga A. Mukhina, Ulyana A. Markina, Yulia G. Alexeeva, Ekaterina A. Nikitina, Marcus Maurer, Alexander V. Karaulov, Maryana A. Lysenko, Thomas Buttgereit

**Affiliations:** 1 GA2LEN Angioedema Center of Reference and Excellence (ACARE), Moscow Research and Clinical Center of Allergy and Immunology, Moscow Healthcare Department, City Clinical Hospital 52, Moscow, Russia; 2 Department of Clinical Immunology and Allergy, The First Sechenov State Medical University, Moscow, Russia; 3 Department of Pulmonology, Astana Medical University, Astana, Kazakhstan; 4 GA2LEN Urticaria Center of Reference and Excellence (UCARE), Institute of Allergology, Charité– Universitätsmedizin Berlin, corporate member of Freie Universität Berlin and Humboldt-Universität zu Berlin, Berlin, Germany; 5 Life Improvement by Future Technologies (LIFT) Center, Skolkovo, Russia; 6 General Therapy Department, Pirogov Russian National Research Medical University, Moscow, Russia; 7 Fraunhofer Institute for Translational Medicine and Pharmacology ITMP, Immunology and Allergology, Berlin, Germany

**Keywords:** Myoferlin, Hereditary angioedema, Angioedema

## Abstract

Data on hereditary angioedema with normal C1 inhibitor levels are currently limited. To date, only one Italian family with HAE-MYOF has been described, comprising exclusively female members. The angioedema (AE) of head and neck area with the teenage onset, triggered by menses and high fever episodes were identified. It is necessary to search for potential biomarkers in patients with HAE-MYOF. This case series reports two unrelated individuals from different families with symptoms onset of recurrent AE and identified myoferlin gene mutations. Due to limited knowledge about the clinical presentation, pathogenesis, and treatment response in HAE-MYOF, further data collection is essential.

Dear Editor,

According to the most recent classification [1], hereditary angioedema (HAE) with a mutation in the myoferlin (MYOF) gene (HAE-MYOF) is categorized as angioedema (AE) caused by intrinsic vascular endothelium (VE) dysfunction (AE-VE). It is hypothesized that the gain-of-function mutation of the myoferlin gene results in increased membrane expression of the vascular endothelial growth factor receptor-2 (VEGFR2), thereby enhancing VEGF signaling and increasing vascular permeability [2]. To date, only one Italian family with HAE-MYOF has been described, comprising exclusively female members [2]. AE of the head and neck area with the teenage onset, triggered by menses and high fever episodes were identified. The search for potential biomarkers of HAE-MYOF is ongoing [3].

This case series reports two independent family’s representatives with symptoms onset of recurrent AE and identified myoferlin gene mutations. Informed consent was obtained from all patients (local ethics committee approval: protocol №10/1023 dated 25.10.2023). ‘The first patient was a 36-year-old male reported the onset of symptoms since the age of 22 with anamnesis of the genital area, tongue, lips, and upper extremities swellings (Fig. 1A). The attacks were preceded by a series of prodromal symptoms, including hyperesthesia, tingling, marginal erythema. In the absence of therapeutic intervention, the AE stayed for a period of up to 72 hours. The average frequency of AE prior to the initiation of therapy was once every 2 months. Injury and pressure were identified as the triggers. No family history of AE was reported. Quantitative and functional levels of C1-inhibitor (C1-INH), as well as C4 were within the normal range. Whole-genome sequencing revealed a newly described heterozygous mutation in exon 33 of the MYOF gene, specifically c.2225C > T(p.Ser742Leu). Variant c.2225C > T(p.Ser742Leu) has not been previously described in the literature. It is not found in the ClinVar database. In gnomADv4.1, it is found only in the Exomes cohort, but not in Genomes. This variant is considered variant of uncertain significance (VUS). Regarding the patient’s father and seven-year-old daughter, further Sanger sequencing did not reveal any mutations in MYOF gene. However, his mother carried the same heterozygous nucleotide substitution in exon 33 of the MYOF gene, c.2225C > T(p.Ser742Leu) asymptomatically. The partial response to on demand treatment with tranexamic acid was observed (resolution of angioedema shortened up to 12 hours), later on the on demand regimen was switched to icatibant. The tranexamic acid 1500 mg/day was initiated as a long-term prophylaxis (LTP) which brought the attack-free period (4 years) with no icatibant need: Angioedema Activity Score (AAS28 0 points), the Angioedema Control Test (AECT, 16 points) and the Angioedema Quality of Life Questionnaire (AE-QoL score, 0 points).

**Fig. 1:**
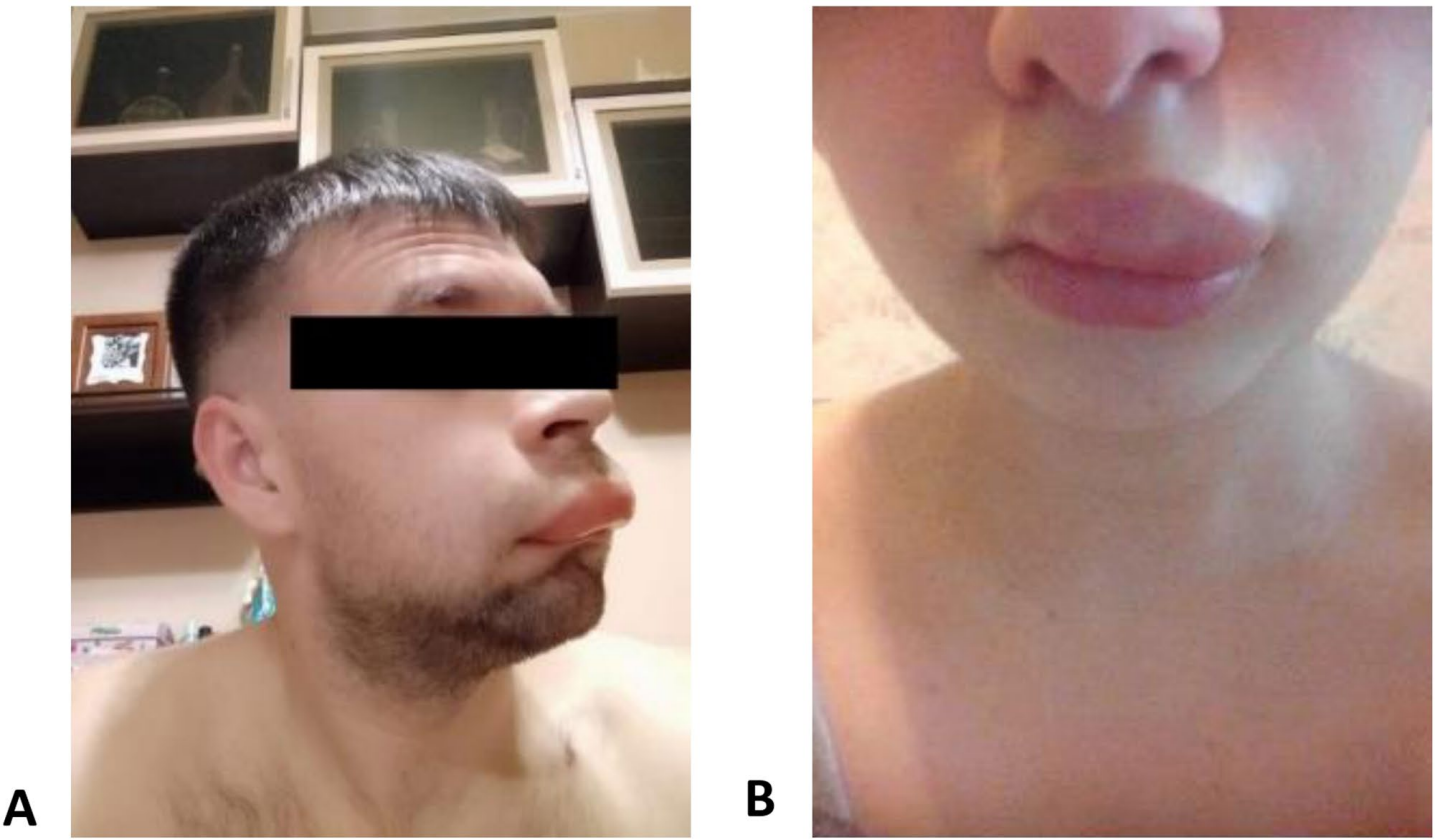
**A** Angioedema of the lips in patient 1 **B** Angioedema of the lips in patient 2

The second family is represented by a 25-year-old female, who has considered herself to be sick since the age of 14, AE appearance in the coccyx area during surgery for a coccygeal cyst. Subsequently, the AE attacks occurred involving eyelids, lips, larynx, pharynx, hands, and feet (Fig. 1B), but no preceding symptoms were observed. In the absence of therapeutic intervention, the AE typically resolved within a period of 72 h with the frequency on average 2–4 times per month. The condition worsened with an increase in the frequency and severity of AE during menses. No additional trigger factors were identified, along with the absence of family history. Quantitative levels of C1INH and C1INH function as well as C4 were within the normal range. Whole-exome sequencing revealed a heterozygous mutation in the MYOF gene, specifically, in exon 33 at position c.3508 C > T(p.His1170Tyr). Variant c.3508 C > T(p.His1170Tyr) has not been previously described in the literature, in ClinVar and in gnomADv4.1 (Exomes + Genomes samples) this variant does not occur and is considered VUS. Long-term prophylaxis with tranexamic acid at a dosage of 1000 mg/day was initiated and the patient was prescribed icatibant for on demand treatment, complete symptom remission was achieved: the AAS28 score was 0 points; the AECT score– 16 points; the AeQoL score– 0 points. Thus there was no need in icatibant as on-demand option. Subsequent examination of the patient’s mother, father, and two sisters for the same mutation using the Sanger sequencing were unremarkable. The patient’s brother, in turn, carries the same mutation (heterozygous mutation c.3508 C > T(p.His1170Tyr) in the MYOF gene) with no clinical manifestations.

Given the lack of knowledge regarding the clinical presentation, pathogenesis, and response to treatment in patients with HAE-MYOF, there is a clear need in data collection. Currently, protein dysfunction assessment is under investigation. Our case series adds at least some more evidence to this rare type of HAE. First, HAE-MYOF has previously been exclusively described in females, however, we present the first case of a symptomatic male patient. And second, this is the first report that describes response to treatment in patients with HAE-MYOF. Interestingly, both patients completely responded to prophylactic treatment with tranexamic acid– a second line treatment option used in patients having bradykinin-mediated HAE, e.g. HAE due to C1-INH deficiency. The mechanisms of effectiveness of tranexamic acid in patients with HAE-MYOF need to be studied. Perhaps this is due to the protective effect of tranexamic acid on the vascular endothelium, resulting in a decreased capillary leakage [4]. At this stage of knowledge the genetic testing has to be accompanied by the search of reliable biomarkers and more studies are needed to assess the response to treatment options.

## Data Availability

The data that support the findings of this study are available from the corresponding author upon reasonable request.

## Abbreviations

HAE: Hereditary angioedema
MYOF: Myoferlin
AE: Angioedema
VE: Vascular endothelium
AE: VE-Angioedema caused by intrinsic vascular endothelium dysfunction
VEGFR2: Vascular endothelial growth factor receptor-2
C1: INH-C1-inhibitor
LTP: Long-term prophylaxis
AAS: Angioedema Activity Score
AECT: Angioedema Control Test
AE: QoL-Angioedema Quality of Life Questionnaire
